# The Impact of Specialized Medical Equipment Needs on Shelter Accessibility for Homeless Patients

**DOI:** 10.7759/cureus.77318

**Published:** 2025-01-12

**Authors:** Juan R Santos-Rivera, Regina J McPherson, Guillermo Izquierdo-Pretel

**Affiliations:** 1 Internal Medicine, Ponce Health Sciences University, Ponce, PRI; 2 Internal Medicine, Florida International University, Herbert Wertheim College of Medicine, Miami, USA

**Keywords:** acute hypercapnic respiratory failure, discharge planning, homelessness, hospital readmission, housing insecurity

## Abstract

Housing insecurity is a well-recognized social determinant of health, with poverty and homelessness significantly impacting health outcomes. When faced with unstable housing, health often becomes a lower priority for patients. We present the case of a 52-year-old homeless, morbidly obese female who arrived at the emergency department with worsening dyspnea. She required Bilevel Positive Airway Pressure (BiPAP) support for acute-on-chronic hypercapnic respiratory failure. Her discharge planning was complicated by the need for a BiPAP machine, which limited her options for placement in a receiving facility. The patient ultimately left against medical advice (AMA) after a seven-day hospitalization but was readmitted hours later with recurrent symptoms, highlighting the ongoing challenges posed by her chronic conditions and social determinants of health. This case highlights how the requirement for specialized medical equipment can serve as an additional barrier to securing shelter for individuals experiencing homelessness.

## Introduction

We define a homeless person as an individual without a stable residence, living either on the streets or in transitional housing [1]. On any given night, approximately 600,000 people in the United States of America (U.S.A.) experience homelessness, with around 400,000 staying in shelters and the remaining 200,000 living on the streets [2]. Several factors contribute to homelessness in the U.S.A., with the primary drivers being the high cost of living, such as rent and groceries and unemployment [3]. While it is important not to generalize about shelters, many of these facilities are not designed for long-term stays. For example, a study conducted in New York and Philadelphia during the 1990s found that about 80% of shelter users stayed for only a brief period [4]. Similarly, a more recent study in Boston (2014-2018) revealed that 21.5% of shelter stays lasted less than 10 days [5].

When seeking shelter, homeless individuals encounter various challenges, both intrinsic and extrinsic. Intrinsic factors, such as feelings of shame or fear, may deter individuals from seeking shelter. Extrinsic barriers may include requirements for documentation, policies excluding pets, or the shelter's inability to accommodate specific needs, such as wheelchair accessibility, special diets, or the need for medical equipment [6]. The discharge process for homeless patients from hospitals is also complicated, as it typically relies on having a fixed address. Homeless patients often face disadvantages when the available shelters cannot meet their medical or personal requirements [7].

A significant concern when discharging homeless patients is ensuring compliance with recommended treatment. Studies show that homeless individuals often have low adherence to treatment, with factors including difficulties in self-managing medications, running out of prescriptions, missing follow-up appointments, medication side effects, and socioeconomic challenges [8]. Moreover, health can become a lower priority when the psychological strain of housing instability takes precedence [9]. To mitigate these negative health outcomes, a collaborative, multisystem approach is essential, focusing on supporting social mobility and reducing housing insecurity.

## Case presentation

In June 2024, a 52-year-old homeless, wheelchair-bound female with morbid obesity presented to the emergency department (ED) with worsening shortness of breath (SOB), fatigue, and syncope twice in one day. She has a past medical history significant for asthma, heart failure with preserved ejection fraction (HFpEF), pulmonary hypertension (HTN), obstructive sleep apnea (OSA), obesity hypoventilation syndrome (OHS), morbid obesity, diverticulitis, and arthritis. Notably, she has a history of incomplete treatment for scabies and is noncompliant with medical therapy secondary to homelessness and lack of resources.

Upon arrival, the patient reported worsening dyspnea. On physical examination, she was morbidly obese but otherwise presented with normal findings except for scattered areas of erythematous papules and scabs concentrated in intertriginous skin areas. She was initially admitted to the ICU due to acute on chronic hypercapnic respiratory failure with worsening PCO₂ levels and increased oxygen requirements despite Bilevel Positive Airway Pressure (BiPAP) therapy.

Hospital course

The patient was diagnosed with acute on chronic hypercapnic respiratory failure and treated with a five-day course of oral antibiotics for suspected infection, budesonide 0.5 mg inhaled twice daily (BID), nocturnal BiPAP therapy with specific settings of 20/8/21 (20 cmH₂O (Inspiratory Positive Airway Pressure or IPAP): Pressure during inhalation to support breathing; 8 cmH₂O (Expiratory Positive Airway Pressure or EPAP): Pressure during exhalation to maintain airway patency and prevent collapse and; 21% FiO₂: Fraction of inspired oxygen, equivalent to room air without supplemental oxygen). These settings were tailored to improve the patient’s ventilation, address hypercapnia, and alleviate respiratory distress due to obesity hypoventilation syndrome (OHS) and obstructive sleep apnea (OSA).

Further diagnostic findings

The Doppler ultrasound test was negative for deep vein thrombosis (DVT). Chest X-ray (CXR) findings revealed mild bilateral interstitial opacities consistent with pulmonary edema or an atypical infectious/inflammatory process, left lower lobe atelectasis/consolidation, and small bilateral pleural effusions greater on the left side. Laboratory test results showed elevated creatinine (1.85 mg/dL), blood urea nitrogen (42 mg/dL), and potassium (5.1 mmol/L) levels.

The patient’s hospital course was complicated by her social situation, particularly her homelessness and need for ongoing BiPAP therapy. Social work was involved to establish stable housing and ensure access to home BiPAP therapy. The receiving facility was a shelter placement, which posed additional challenges for the patient's continued care. After seven days of hospitalization, the patient left against medical advice (AMA). However, she was readmitted a few hours later with similar complaints of acute chronic dyspnea.

## Discussion

**Figure 1:**
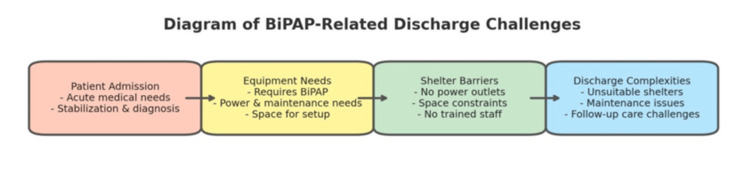
Barriers to discharge homeless patients requiring BiPAP therapy This diagram illustrates the challenges associated with Bilevel Positive Airway Pressure (BiPAP)-related discharge planning for patients experiencing housing insecurity. It outlines key barriers, including medical stabilization, equipment needs, shelter limitations, and discharge complexities, highlighting the interplay between medical care and social determinants of health. The author Juan R. Santos Rivera directed the creation of this image using ChatGPT-4o.

Discharging a homeless patient often presents numerous challenges, particularly regarding treatment adherence and finding suitable placements that meet their medical and social needs. One of the primary reasons for non-adherence to treatment among homeless individuals is socioeconomic constraints [8]. This case highlights an often-overlooked barrier to securing appropriate placement: the need for specialized medical equipment. As illustrated in Figure 1, the need for medical equipment creates an additional barrier to finding shelter. Homeless individuals requiring medical equipment, such as BiPAP, face additional challenges when seeking shelter. Many shelters lack the necessary infrastructure and space to support medical devices (6). Additionally, shelter staff often lack the training to assist with the maintenance or troubleshooting of BiPAP machines, limiting their viability in this environment (7). Financial constraints, liability concerns, and even noise complaints from other residents may all contribute to policy restrictions on medical equipment (8). Addressing these obstacles will require collaboration across multiple sectors to improve facilities, funding, and policies. The patient in question faced an unfair dilemma between receiving the necessary treatment for her condition or obtaining shelter. 

This situation underscores the risk of increased readmission rates due to non-compliance with essential therapy. To break this cycle, we must address the socioeconomic realities faced by homeless patients and implement solutions that cater to their specific needs. While healthcare systems are already under significant strain, with readmission rates being a key pressure point, a multidisciplinary approach is crucial. Equipping shelters with the necessary medical tools and resources to accommodate patients is one potential solution. 

Although community-based support systems exist to assist homeless individuals and those facing job insecurity, these frameworks require restructuring to tackle broader social issues, such as family stability, early childhood development, and education-that ultimately influence housing insecurity [10]. Given the strong association between homelessness and poorer health outcomes, addressing housing insecurity should be a priority for preventive strategies aimed at improving affordability and stability. Doing so would not only reduce healthcare costs but also enhance overall community health [11].

Despite the limited number of studies on this topic, it is clear that homelessness presents ongoing challenges that demand urgent attention. This case emphasizes the need for a sustained, integrated, and interdisciplinary approach to addressing both the health and social needs of this population [12]. These two aspects are deeply interrelated and should not be treated in isolation.

## Conclusions

Homeless patients represent a unique population that poses multiple challenges during hospital discharge planning. Various barriers to placement can negatively affect compliance with prescribed therapies and increase the risk of readmission. Discharging the patient was particularly difficult due to the need for a BiPAP machine, which added an underappreciated obstacle to placement. This case presents an additional neglected obstacle for homeless individuals who require specialized medical equipment, such as a BiPAP machine, upon discharge. For this vulnerable population, it is essential that shelters expand their services to include support for medical equipment. Possible solutions may include creating specialized medical shelters, shelter-healthcare collaborations, policy changes, or funding for equipment support. These solutions aim to provide basic medical services and support for essential equipment, such as BiPAP, to ensure that homeless individuals have access to both safe housing and necessary care. Our aim is to raise awareness of this underrepresented barrier and advocate for more comprehensive solutions to ensure better outcomes for homeless patients.
